# Identification and Characterization of a *Cis*-Encoded Antisense RNA Associated with the Replication Process of *Salmonella enterica* Serovar Typhi

**DOI:** 10.1371/journal.pone.0061308

**Published:** 2013-04-23

**Authors:** Isaac Dadzie, Shungao Xu, Bin Ni, Xiaolei Zhang, Haifang Zhang, Xiumei Sheng, Huaxi Xu, Xinxiang Huang

**Affiliations:** 1 Department of Biochemistry and Molecular Biology, School of Medical Science and Laboratory Medicine, Jiangsu University, Zhenjiang, Jiangsu, China; 2 Department of Laboratory Technology, University of Cape Coast, Cape Coast, Ghana; Beijing Institute of Microbiology and Epidemiology, China

## Abstract

Antisense RNAs that originate from the complementary strand of protein coding genes are involved in the regulation of gene expression in all domains of life. In bacteria, some of these antisense RNAs are transcriptional noise whiles others play a vital role to adapt the cell to changing environmental conditions. By deep sequencing analysis of transcriptome of *Salmonella enterica* serovar Typhi, a partial RNA sequence encoded in-*cis* to the *dnaA* gene was revealed. Northern blot and RACE analysis confirmed the transcription of this antisense RNA which was expressed mostly in the stationary phase of the bacterial growth and also under iron limitation and osmotic stress. Pulse expression analysis showed that overexpression of the antisense RNA resulted in a significant increase in the mRNA levels of *dnaA*, which will ultimately enhance their translation. Our findings have revealed that antisense RNA of *dnaA* is indeed transcribed not merely as a by-product of the cell's transcription machinery but plays a vital role as far as stability of *dnaA* mRNA is concerned.

## Introduction

Many non-coding RNAs (ncRNAs) in bacteria have been identified in recent times to adapt the bacteria to changing environmental conditions as well as influencing their virulence. Majority of the ncRNAs characterized so far act by base pairing with target mRNAs to either modulate the stability of the mRNA or block or promote ribosome binding to mRNAs to alter translation processes [1]. Two classes of bacterial ncRNAs can be distinguished: *trans-*encoded and *cis*-encoded. *Trans*-encoded RNAs are located in another chromosomal location, and are only partially complementary to their target RNA(s), whereas the less focused on *cis*-encoded antisense RNAs (asRNAs) are located in the same DNA region and are, therefore, fully complementary to their targets over a large nucleotide stretch [2].

The chromosomally encoded asRNAs differ in size as well as region of overlap between them and their target mRNAs. Many are substantially long, ranging from 700 to 3,500 nt, although some are only 100 nt in size. These asRNAs can also overlap the 5′-end, the 3′-end, the middle or the entire gene encoded opposite [3], [4]. The known regulatory mechanisms employed by *cis*-encoded asRNAs include transcription attenuation, translation inhibition, inhibition of primer maturation, promotion or inhibition of mRNA degradation and prevention of RNA pseudoknot formation [4], [5]. AsRNAs are involved in a number of cellular processes in bacteria, including acid resistance [6], iron homeostasis [7], quorum sensing [8], Mg^+2^/Ca^+2^ transport and virulence [9]–[11], ABC transport systems [12], global repression of OMP synthesis [13], [14] and control of expression of global transcription factors [15], [16].

Second generation sequencing analysis we conducted to find out the involvement of *cis-*encoded antisense RNAs in bacterial replication identified, among others transcripts of antisense RNA of *dnaA*. DnaA is the bacterial replication initiation factor, a site-specific DNA binding protein that recognizes the origin of replication (*oriC*) and initiates the assembly of the DNA replication machinery. The *oriC* contains an asymmetric 9-bp recognition sequence, the DnaA box: 5′-TTATNCACA, which are sequence-specific binding sites for the initiator protein DnaA [17], [18]. The interactions of DnaA with *oriC* mediates open complex formation and allows assembly of an initiation complex that loads the replicative helicase and facilitate the recruitment of the remaining replisome components, leading to replication of the bacteria [19]. DnaA also functions as a transcription factor, recognizing specific promoters and activating or repressing the transcription of target genes [20], [21]. In *E. coli* prokaryotic model, systems that regulate DnaA function have been established. These include the titration of DnaA to a specific site termed *datA* locus, the repression of *dnaA* transcription immediately after replication by the activity of SeqA, the regulatory inactivation of DnaA (RIDA) system which promotes ATP hydrolysis in a replication-coupled manner to yield initiation-inactive ADP–DnaA, and the transcriptional autoregulation through the binding of DnaA to DnaA boxes in the promoter region, which prevents an over-abundance of DnaA and additional initiation events [18], [22].

Although recent application of advanced sequencing technologies has revealed that chromosomal transcription of antisense sequences is pervasive in bacteria, the functional relevance of most of the antisense transcripts is unknown [3], [4]. In this paper, we describe the identification and characterization of a *cis*-encoded antisense RNA of *dnaA* of *Salmonella enterica* serovar Typhi (*S*. Typhi), which we termed AsdA (antisense RNA of *dnaA*) and demonstrate that the expression of this antisense RNA increases the stability of *dnaA* mRNA which ultimately have a concomitant effect on the level of DnaA.

## Materials and Methods

### Bacterial strains, plasmids, and growth conditions

Bacteria were routinely grown at 37°C in Luria–Bertani (LB) medium with or without ampicillin (Amp) at a concentration of 100 µg/ml. All strains used are derivatives of *S*. Typhi GIFU10007 z66-positive wild-type and are listed in Table 1. The oligonucleotides used in this study can be found in Table 2. To construct pBAD*-asdA*, a *Nco*I*-Hind*III 623 bp fragment which corresponds to the 540 bp *asdA* sequence and 83 bp downstream of *asdA* was amplified by PCR with primers asdA-F and asdA-R using genomic DNA from the GIFU10007 strain as template. This was subcloned into *Nco*I*-Hind*III sites of pBAD/Myc-His A (Invitrogen). pBAD-*asdA*96 was also constructed by ligating a *Nco*I*-Hind*III fragment made up of 141 bp 5′ sequences of *asdA* to *Nco*I*-Hind*III *sites of* pBAD Myc-His A. The wild type strain was then transformed by electroporation with pBAD*-asdA*, pBAD-*asdA*96 and pBAD empty plasmid to obtain strains designated as 007-*asdA*, 007-*asdA*96 and 007-pBAD respectively. DNA sequencing verified the presence of the inserts. To construct RNase III mutant strain (Δ*rnc*), primer pairs RIII-F1A/RIII-F1B and RIII-F2A/RIII-F2B were used to amplify the fragments F1 (419 bp) and F2 (582 bp) located upstream and downstream of the RNase III gene, respectively. A *Bam*HI site was added to the 5′-termini of primers RIII-F1A and RIII-F2B. Primers RIII-F1B and RIII-F2A share ten base overlapping complementary sequences. The PCR amplicons of F1 and F2 fragments were used as template for a second PCR reaction using primers RIII-F1A and RIII-F2B to obtain a single 1001 bp fragment without 366 bp sequences of *rnc* gene. The 1001 bp fragment was digested with *Bam*HI restriction enzyme and inserted into *Bam*HI site of the suicide plasmid pGMB151, which carries a sucrose-sensitivity *sacB* gene. Suicide plasmid with the insert was electroporated into the *S.* Typhi wild type strain. RNase III mutant strains were selected on LB plates with sucrose and inserts were verified by PCR with primers RIII-F1A and RIII-F2B and sequencing. RNase E mutant (Δ*rne*) was also constructed as described above using primer pairs RE-F1A/RE-F1B and RE-F2A/RE-F2B. pBAD*-asdA* was electroporated into the RNase III and RNase E mutants to obtain Δ*rnc*007-*asdA* and Δ*rne*007-*asdA*, respectively. Fur deletion mutant was constructed using the lambda red recombinase method [23]. A Kanamycin-resistant gene was amplified from plasmid pKD46 with primers FUR-FA/FUR-FB, which apart from the region of complementarity with the *kan* sequences they were flanked by 41 bp of *fur* sequences. Purified PCR product was used to transform the GIFU10007 containing plasmid pKD46.

**Table 1:** Strains and plasmids used in this study.

Strains and plasmids used in this study	Relevant characteristics	Source
**Strains**		
S. Typhi GIFU10007	wild-type strain of S. Typhi; z66+	[40]
Δ*rnc*007-*asdA*	GIFU10007 (Δ*rnc*), z66+ containing pBAD-*asdA*	This work
Δ*rne*007*-asdA*	GIFU10007 (Δ*rne*), z66+ containing pBAD-*asdA*	This work
SY372*λpir*	suicide plasmid *E. coli* host	[40]
TOP10	*recA, endA E. coli* host	Invitrogen
007-pBAB	GIFU10007 containing pBAD Myc-His A empty plasmid	This work
007-*asdA*	GIFU10007 containing pBAD-*asdA*	This work
007-*asdA*96	GIFU10007 containing pBAD-*asdA* 96	This work
Δrnc-pBAD-asdA	Δ*rnc* with pBAD containing *asdA* DNA	This work
Δ*fur*	GIFU10007 (Δ*fur*),	This work
Δ*rpoS*	GIFU10007 (Δ*rpoS*),	[41]
**Plasmids**		
pGMB151	Suicide plasmid; *sacB*; Amp^r^	[40]
pBAD Myc-His A	A pBAD expression plasmid	Invitrogen
pBAD/gIII	Expression vector; Amp^r^	Invitrogen
pGEM-T	*E. coli* TA cloning vector; Amp^r^	Promega
pBAD-*asdA*	pBAD plasmid expressing *asdA* (a 623 bp fragment antisense to *dnaA*)	This work
pBAD-*asdA*96	pBAD plasmid expressing the short, predominant 96 bp fragment of *asdA*	This work

**Table 2:** Oligonucleotides used in this study.

Primers	Sequence in 5′→3′ direction
**Primers and adaptor used for 5′-and-3′ RACE**	
5-R1	CAGACAACGACGGCGCAA
5-R2	CTACCGCTCCAACGTCAA
asdA -qR	CAGACAACGACGGCGCAA
5-ASP-1	CATGGCTACATGCTGACAGCCTA
5-ASP-2	CGCGGATCCACAGCCTACTGATGATCAGTCGATG
3-ASP	GGCCGCTAAGAACAGTGAA
3-AD	5′-phosphate-UUCACU GUUCUU AGC GGC CGC AUG CUC-idT -3
**Primers used for Real-time PCR analyses**	
dnaA-qR	CAGCAGAGCGTTAAAGGTGT
dnaA-qF	CGCAAACCCAACGCGAAAGT
asdA –qR	CAGACAACGACGGCGCAA
asdA –qF	TTGACGTTGGAGCGGTAG
5S-qR	TTTGATGCCTGGCAGTTC
5S-qF	TTGTCTGGCGGCAGTAGC
**Primers used to construct strains**	
asdA-F	TGACCATGGGGTGCCGCCATAGAGGAATA
asdA-R	TAGAAGCTTCCTGTGGATAAATCGGGAAG
asdA-96	TCTAAGCTTTACCGCTCCAACGTCAATGT
RIII- F1A	TTTGGATCCCGTATCGGCGGTATTCACTA
RIII- F1B	GCAAATACTCGCGTTCGTTATGTTTACTGC
RIII-F2A	TAACGAACGCGAGTATTTGCAGGGTCGCCA
R-III F2B	TTTGGATCCGCACGTTATCCACCTTG TTA
RE-F1A	TAAGGATCCAGTGGAATAATGATGCGGT
RE-F1B	GATCGTCAACAATACGGGTAATTTTGCCT
RE-F2A	TACCCGTATTGTTGACGATCATAACCACG
RE-F2B	TGAGGATCCGATCTCATAAACGCCTGGA
FUR-FA	CTCTAATGAAGTGAATCGTTTAGCAACAGGACAGATTCCGCGGTCTGACGCTCAGTGGA
FUR-FB	AAAAAGCCAACCGGGCGGTTGGCTCTTCGAAAGATTTACACTTTCGGCCTATTGGTTAA
**Probes used for Northern blot**	
asdA-PB	GATAATCCTGGCGGCGCCTATAACCCGTTATTCCTCTATG
asdA -nF	TGCCGCCATAGAGGAATAAC
asdA -nR	AATTGTAATACGACTCACTATAGGGCGGTTCGGGCTGGGATAACG

### RNA extraction

Overnight cultures of *S*. Typhi wild type strain and mutants were diluted 1/100 in LB medium and grown at 37°C with shaking (250 rpm). To determine the expression of *asdA* at different time phase, samples were taken at OD_600_ values of 0.3, 0.8, 1.3 and 2. To determine the expression of *asdA* under different stress conditions, bacteria were grown to OD_600_ 0.8 and treated with 0.5 M NaCl, representing osmotic stress, 1 mM hydrogen peroxide for oxidative stress, 0.2 mM 2,2,-dipyridyl for iron limitation. For acid stress, the pH of the culture medium was adjusted (with HCl) to 4.5. The bacteria cells were then grown for an additional 30 min after stress induction. To carry out over expression analysis, overnight cultures of *S*. Typhi carrying an empty pBAD Myc-His A plasmid (007-pBAD) or plasmid expressing *asdA* (007-pBAD-*asdA* and 007-*asdA*96) were diluted 1/100 in LB medium and grown at 37°C to OD_600_ 0_._6. Expression of *asdA* was induced by the addition of 0.02% of L-arabinose. Aliqoutes were taken prior to or at 5, 10 and 20 min after L-arabinose addition. To extract total RNA, the cultures were pelleted by centrifugation at a speed of 16,000 g for one minutes and RNA was isolated using Trizol (Life Technologies). RNA samples were treated with DNase I (Takara) to eliminate DNA contaminations and purified RNA was quantified using a ND-100 Spectrophotometer (NanoDrop Technologies).

### 5′-and 3′-RACE

5′-RACE (rapid amplification of cDNA ends) was carried out with the 5′-Full RACE kit (Takara) according to the manufacturer's instructions. Briefly, 5 µg of total RNA preparation was treated with 10 unit of calf intestine alkaline phosphatase (CIAP) for 1 hour at 50°C to exclude processed or decayed target RNAs. 5′-triphosphates were converted to monophosphates by treatment of CIAP-treated RNA with 1 unit of tobacco acid pyrophosphotase (TAP) for 1 hour at 37°C. The CIAP/TAP-treated RNA was ligated to 250 pmol of the supplied 5′-RACE adaptor with 40 unit of T4 RNA ligase for 1 hour at 16°C. Reverse transcription (RT) was carried out at 42°C for 1 hour with 5 U M-MLV reverse transcriptase and 25 pmol of antisense RNA specific primer. All reactions were performed in the presence of 10 U RNase inhibitor. One microliter of the resulting cDNA was amplified with 25 pmol of 5′-RACE adaptor specific primer (5-ASP-1) and *asdA* specific primer (5-R1). A second amplification was performed with 5-ASP-2 and 5-R2 primers using product of the first PCR as template. Purified PCR products were cloned into pGEM-T vector (Invitrogen). Bacterial colonies obtained after transformation were screened for the presence of appropriate inserts by PCR and confirmed by sequencing. 3′-RACE experiments were carried as described previously [24]. Total RNA (15 µg) was dephosphorylated with calf intestine alkaline phosphatase (Takara). Phenol-chloroform extracted and ethanol precipitated RNA was ligated to 5′-phosphorylated 3′ RACE adaptor (3-AD). Reverse transcription was performed as described for 5′-RACE with 200 pmol of adaptor specific primer (3-ASP) complementary to 3-AD and *asdA* specific primer (asdA-qF). PCR amplification, cloning, and sequence analysis was done as described above.

### Quantitative RT-PCR

Four microgram (4 µg) of DNase I treated total RNA was used for cDNA synthesis using Super Script III reverse transcriptase (Invitrogen) and gene specific primers according to the manufacturer's protocol. Quantification of cDNA was performed using SYBR Premix Ex Taq II (Takara) and appropriate primers (*dnaA*: dnaA-qR/dnaA-qF; *asdA*: asdA-qR/asdA-qF) and monitored using C1000 Thermal Cycler (Bio-Rad) according to manufacturer's instructions. Relative RNA levels were determined using the comparative CT method [25]. In order to confirm that there was no DNA contamination, a negative control was included in each run. Three independent sets of experiments were performed. All samples were normalized against levels of 5S ribosomal RNA amplified with primer pairs 5S-qF/5S-qR.

### Northern blot analysis

For *asdA* RNA detection, 5–20 µg of total RNA was separated on 7M urea/6% polyacrylamide gels in 1× TBE and electrobloted to Hybond-XL membranes. Riboprobes were synthesized with primer pairs asdA–nF/asdA–nR using DIG Northern Starter Kit (Roche) following the manufacturer's protocol. Following pre-hybridization of the membranes in Rapidhyb buffer (GE Healthcare), or Hyb hybridization buffer (Innogent), membranes were hybridized overnight at 68°C with DIG-labeled riboprobes, or at 42°C in the case of ^32^P-ATP labeled asdA-PB oligoprobes. After hybridization, membranes were washed as described [26]. Riboprobed membranes were immunologically detected while oligoprobed membranes were exposed to KODAK Biomax XAR film at −70°C.

### Growth curves

Growth of strains was determined by measured OD using a BioPhotomerter (Eppendorf). Single colonies of 007-pBAD, 007-*asdA* and 007-*asdA*96 strains were cultured overnight at 37°C with ampicillin (100 µg/ml) and diluted 1/100 in LB medium with 0.02% L-arabinose. The growth rate under normal conditions was determined by growing cells at 37°C with shaking and absorbance (OD_600_) taken at 1 hour intervals for 14 hours. To determine the growth rate under stress conditions, cells were grown at 37°C with shaking for four hours and treated with either HCl to a pH of 4.5, 1 mM of H_2_O_2_, 0.3 M NaCl or 0.2 mM of 2, 2-dipiridyl, representing acid stress, oxidative stress, osmotic stress and iron limitation, respectively. Each growth curve was performed in biological triplicate.

## Results

### Mapping of the 5′ and 3′ ends of *asdA*

**Figure 1:**
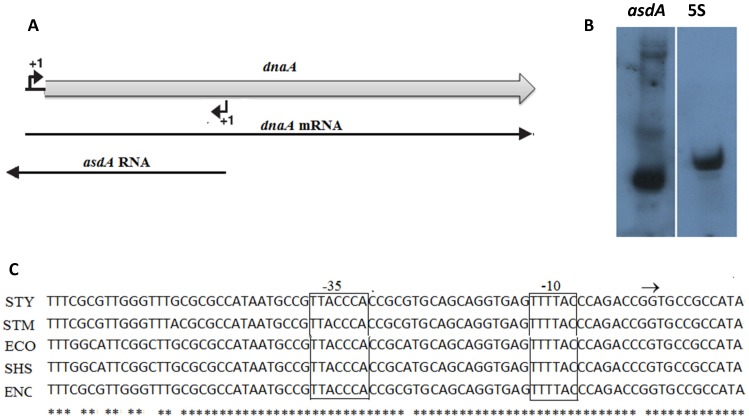
Expression of cis-encoded antisense of *dnaA.* A. The antisense RNA (*asdA*) encoded by the *dnaA* gene. B. Northern blot analysis of RNA isolated from wild-type S. Typhi grown to OD_600_ 1.3 and probed with riboprobes obtained using the primer asdA-nF/asdA-nR. C. Alignment of *asdA* sequences showing the conservation of the promoter region. The transcription start site is indicated by an arrow and the −10 and −35 promoter elements are boxed. The asterisk (*) indicates the highly conserved sequences among various Enterobacteria. Abbreviations for bacterial species names are: *Salmonella* Typhi (STY), *Salmonella* Typhimurium (STM), *Escherichia coli* (ECO), *Shigella sp* (SHS), *Enterobacter sp* (ENC).

We determined the boundaries of *asdA* by 5′-and 3′-RACE analysis. 5′-RACE for mapping of transcription start sites produced a single 5′-end, located 514 nucleotides downstream of *dnaA* start codon. 3′-RACE mapping of transcription termination point detected three different 3′-ends in several independent clones. One site which was detected in all the clones is located 61 nucleotides downstream of the *dnaA* start codon whereas the other 3′-ends are present 295 and 423 nucleotides downstream of *dnaA* start codon. The three transcripts obtained may be as a result of endonucleotic activity of RNases on the full transcript of *asdA.* We conducted northern blot with riboprobes generated from primer pairs asdA-nF/asd-nR to confirm the expression of *asdA*. The blot as shown in figure 1B detected four different bands, three of which corresponded in size with results obtained by RACE. The fourth band which is approximately 540 nucleotides long (from northern analysis) could not be detected after a series of 3′-RACE experiments. It could also be noted that the short 96 nucleotide band was the predominant and the most expressed transcript of *asdA* (figure 1B). Putting the results of RACE and Northern blot together the full length of *asdA* was estimated to be 540 nucleotides long.

Only a small fraction of asRNAs are conserved across species [27] and in view of the fact that DnaA and its binding sites are well conserved throughout the bacterial kingdom [28], we compared the promoter regions of *asdA* with that of other *Enterobacteria*. As shown in figure 1C, it is obvious that the antisense transcripts of *dnaA* are conserved in species of *Salmonella, Escherichia, Enterobacter* and *Shigella*.

### Analysis of *asdA* expression under different growth conditions

**Figure 2:**
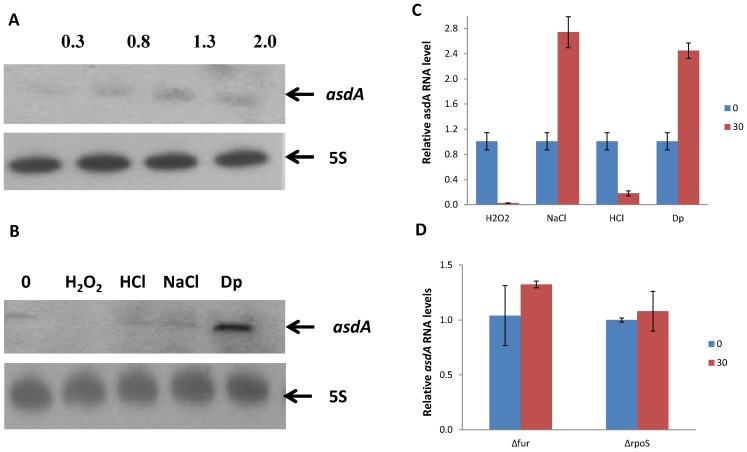
Expression of *asdA* under different growth conditions. A. Northern blot analysis performed on total RNA isolated from *S.* Typhi cultures at different OD_600_ values, as shown across the top of the blots. 5S rRNA was used as loading controls. B. Northern blot analysis of total RNA isolated from *S*. Typhi cells grown in LB to OD_600_ value of 0.6 and subjected for 30 min to osmotic shock (NaCl: 0.5 M), oxidative stress (H_2_O_2_: 1 mM hydrogen peroxide), low iron conditions (Dp: 0.2 mM 2,2,-dipyridyl), acid stress (HCl: pH 4.5). Northern blot was performed with oligoprobe asdA-PB. C. qRT-PCR analysis of total RNA isolated from *S*. Typhi cells and subjected to the same conditions as described in B above. D. qRT-PCR analysis of RNA extracted from *fur* and *rpoS* mutant *S*. Typhi strains under iron limitation and osmotic stress respectively.

To gain insight into the expression of *asdA*, we carried out qRT-PCR and Northern blot analysis with RNA harvested from the wild-type strain grown in LB at different time phase and stress conditions. To determine the expression of *asdA* at different time, RNA was extracted at OD_600_ of 0.3, 0.8, 1.3, and 2.0 representing the growth phases of bacteria from the lag phase through to the stationary phase. Highest level of expression was observed at the stationary phase (figure 2A). To determine the expression of *asdA* under stress conditions, total RNA was extracted after cells were subjected to acid stress, oxidative stress, iron limitation and osmotic shock, which are some of the conditions reminiscent of the environment *Salmonella* encounters upon invasion or within macrophages. qRT-PCR result (figure 2C) shows more than twofold increase in expression of *asdA* upon osmotic stress and iron limitation, however upregulation of *asdA* was observed only under iron limitation when Northern blot analysis was conducted. The above results are consistent with the observation that most non-conding RNA transcription has been shown to be in response to specific growth and environmental/stress conditions [29], [30].

Considering the fact that a large number of different transcriptional regulators have been found to regulate ncRNAs, we assessed the transcription of *asdA* under iron limitation and osmotic stress in Fur and RpoS mutant strains respectively. As shown in figure 2D, the relative amount of AsdA before and after stress induction was virtually the same in both strains, an indication that both the iron-responsive Fur regulator and the stress sigma factor (RpoS) may be involved in the transcriptional regulation of *asdA* under their respective conditions.

### Effect of overexpression of *asdA* on *dnaA* mRNA level

**Figure 3:**
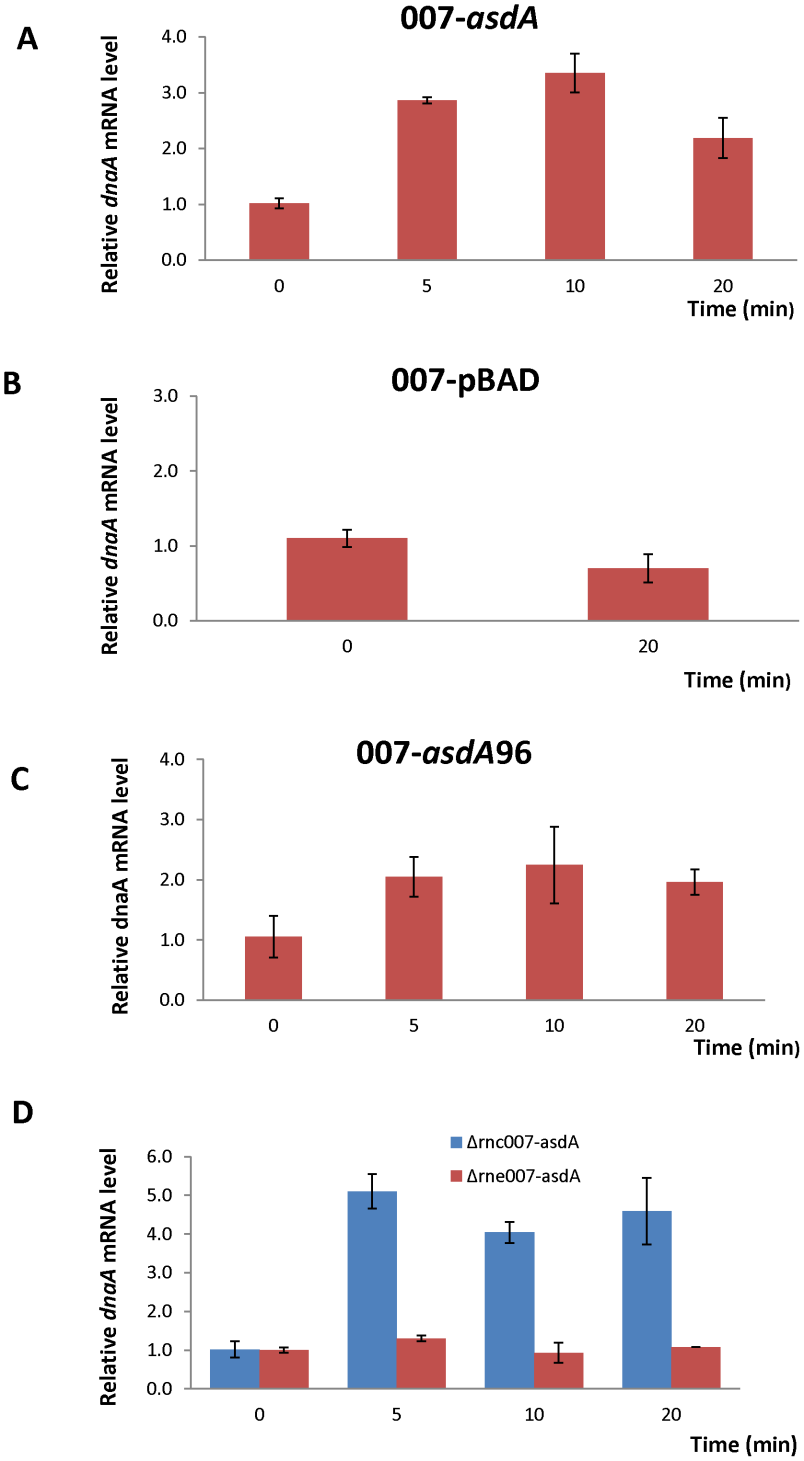
Overexpression analysis of *asdA*. qRT-PCR results of *dnaA* mRNA levels in A. 007-*asdA*, B. 007-pBAD, C. 007-*asdA*96 and D. Δrnc007*-asdA* and Δrne007-*asdA* strains. Total RNA was isolated from these strains grown to an OD_600_ of 0.6 at 0, 5, 10, and 20 min after addition of L-arabinose (0.02% final concentration) to cultures.

We monitored the levels of *dnaA* mRNA by qRT-PCR upon full length overexpression of *asdA* from arabinose inducible pBAD promoter. As shown in Figure 3A, the mRNA level of *dnaA* was significantly higher when *asdA* is overexpressed as compared with a wild type strain carrying an empty pBAD plasmid (figure 3B). As stated above, the short 96 nucleotide transcript of *asdA* was the most highly expressed and the most stable. We thus constructed a wild type strain carrying pBAD plasmid expressing the 96 nucleotide antisense truncated transcript and tested its effect on mRNA levels of *dnaA* when it is overexpressed. Figure 3C shows that the short, truncated transcript was enough to cause a significant increase in *dnaA* mRNA level when it is overexpressed, and also shows similar expression pattern as the full length *asdA*.

RNase III and RNase E are the two major endoribonucleases that have been linked to antisense RNA-induced target mRNA cleavage. RNase III cleaves double-stranded RNA whiles RNase E is a single stranded-specific endoribonuclease and also serves as the scaffold for the other protein components in the degradosome assembly [31], [32]. We constructed strains expressing *asdA* from arabinose inducible promoter in *rnc* and *rne* mutant background to determine the effect of *asdA* overexpression on the target mRNA level in these two RNase mutants. mRNA levels of *dnaA* remained relatively the same in RNase E mutant whiles it increased up to 5 fold in RNase III mutant (figure 3D), giving an indication that RNase III may be involved in the degradation of *asdA*/*dnaA* RNA duplex.

### Effect of overexpression of *asdA* on the growth of *S.* Typhi

**Figure 4:**
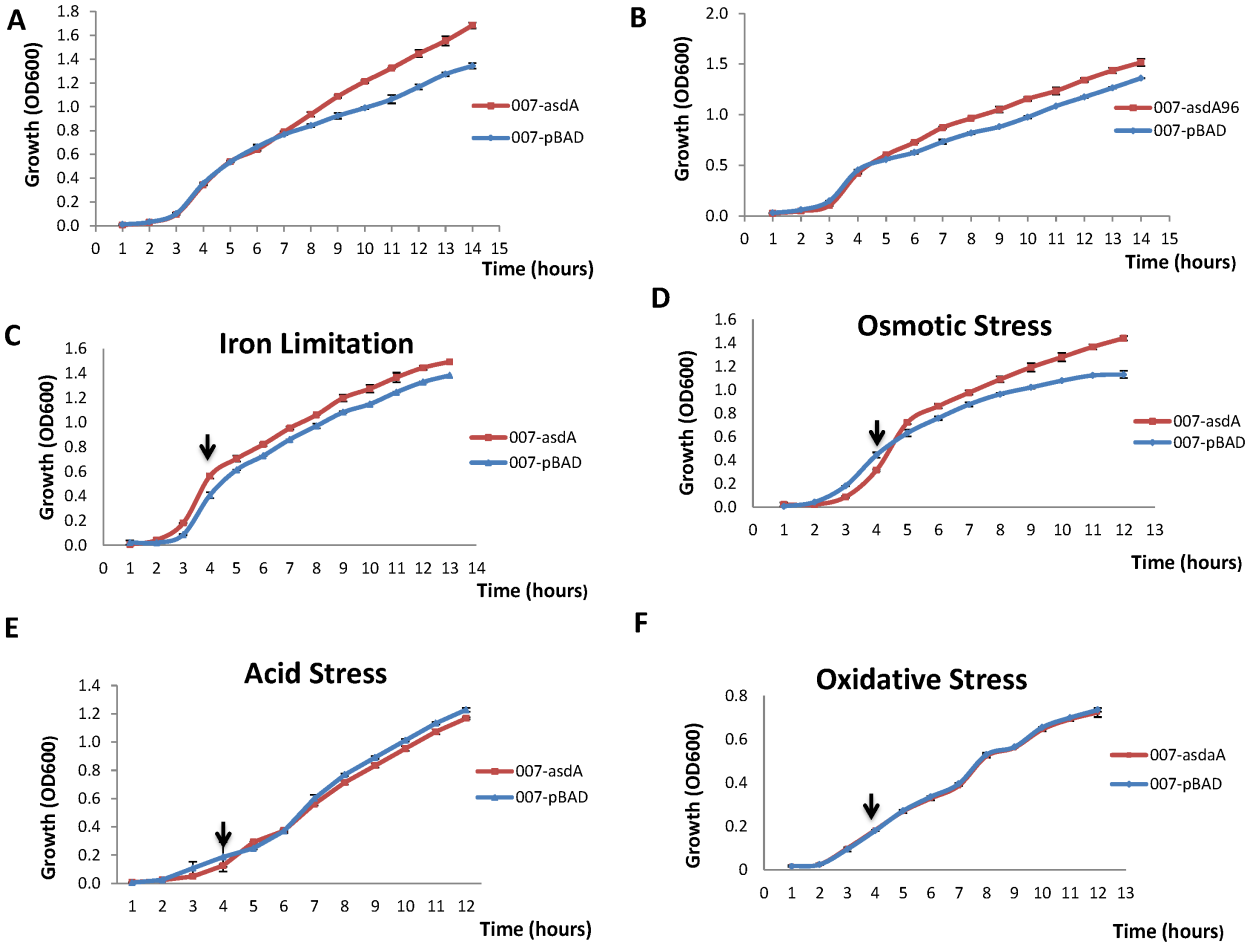
Growth curves of 007-pBAD, 007-*asdA* and 007-*asdA*96 strains. Single colonies of cells were cultured overnight at 37°C with ampicillin (100 µg/ml) and diluted 1/100 in LB medium with 0.02% L-arabinose. Cells were then grown at 37°C with shaking. Growth curve was determined under normal conditions (A and B) by taking the absorbance (OD_600_) at 1 hour intervals for 14 hours. For growth under stress conditions, cells were grown for four hours and treated with either HCl to a pH of 4.5, 1 mM of H_2_O_2_, 0.3 M NaCl or 0.2 mM of 2, 2-dipiridyl, representing iron limitation (C), osmotic stress (D), acid stress (E) and oxidative stress (F) respectively. Absorbance was read at 1 hour intervals for additional 8 hours. Arrows indicate the time point at which stress conditions were induced.

We monitored the growth of the wild type strain containing an empty pBAD plasmid (007-pBAD) or plasmid expressing *asdA* (007-*asdA*) over a fourteen hour period. As shown in Figure 4A, the growth rate of the two strains was similar during the lag and exponential phase. However approaching the stationary phase, the strain overexpressing *asdA* grew better than that with empty plasmid. This pattern of growth was also observed for the strain overexpressing the short truncated transcript of *asdA* (figure 4B), confirming the fact that this short transcript exerts similar effect as the full length. The growth rate was also determined for the 007-pBAD and 007-*asdA* strains under selected stress conditions. As shown in figure 4C and D, the growth of the two strains was similar under oxidative and acid stress however, 007-*asdA* grew better than that of 007-pBAD under iron limitation and osmotic stress.

## Discussion

Bacterial ncRNAs are key players in reprogramming protein expression upon environmental change, in particular under stress conditions. The *trans*-encoded RNAs are the most extensively studied of the known non-coding RNAs. Although chromosomal antisense transcription has been shown to be widespread in bacterial genomes [27], [33], [34], it has attracted less attention and so the conditions that govern the synthesis of these antisense RNAs, as well as their physiological role and mechanism of action remain largely unknown. We report in this study the discovery of a *cis*-encoded RNA that is involved in bacterial replication.

To confirm the expression of *asdA* we performed northern blot and RACE analysis. The result of both analysis indicated extreme processing of AsdA by RNases, leading to multiple bands seen in the northern blot and different 3′-ends in the RACE analysis. The length of *asdA* indicates that it has full complementarity with one third of the coding sequences of *dnaA*, from nucleotide 884 upstream of the stop codon extending through to about 25 bases upstream of the start codon of *dnaA* (figure 1A). We noticed that results of the riboprobed northern blot, RACE and the transcription signals of *asdA* detected by deep sequencing indicated a shorter transcript which was the most stable and highly expressed. It is possible that the increased levels of *dnaA* mRNA observed in other experiments (discussed below) may be mainly due to the expression of this transcript.

Alignment of sequences of antisense strands of *dnaA* gene shows that they are highly conserved at the promoter region and the transcription start sites in species of *Salmonella, Escherichia, Shigella and Enterobacter* (figure 1C). Studies conducted by Raghavan et al [27] to investigate the biological relevance of antisense transcripts concluded that asRNA promoters show no evidence of sequence conservation between, or even within, species and that many or even most bacterial asRNAs are nonadaptive by-products of the cell's transcription machinery. Given that the degree of conservation among homologous sequences provides an effective method for discriminating functional from nonfunctional sequences, we may speculate that the likelihood of antisense transcripts of *dnaA* being expressed and playing a role in other *Enterobacteria* is very high.

Expression of *asdA* RNA was highest during the stationary growth phase and under two stress conditions (figure 1). Monitoring the growth rates of both the wild strain of *S*.Typhi and strains overexpressing *asdA*, be it the full length or the short predominant transcript, reveals that expression of asRNA enhances growth. It could be observed that growth of the wild type and AsdA overexpressing strains were similar during the lag phase and the early exponential phase (figure 3A and B). However, the growth of the latter was enhanced during the late exponential and stationary phase, where endogenous transcription of AsdA was highest. It could also be seen that under iron limitation (figure 3C) and upon induction of osmotic stress (figure 3D), Asda overexpressing strains grew better than the wide type. AsdA was upregulated under these stress conditions (figure 2B and C). AsdA was downregulated under acid and oxidative stress, whiles the growth of both strains used in this study was also similar under these conditions. Results obtained are in conformity with the general observation that transcription of non-coding RNAs in general is activated in response to specific growth and stress conditions and their activities aid cells in recovery from those stresses [2].

ncRNAs have been assigned to various important regulons of *E. coli* and *Salmonella.* For instance, MgrR and AmgR are regulated by the PhoQ/P two-component system (TCS) [35], [36], CyaR transcription is controlled by the cAMP–CRP complex [37] and MicA and RybB are activated by the envelope stress sigma factor (σ^E^) [38], [39]. Fur protein is a key regulator of iron metabolism whereas the activity of stress sigma factor (RpoS) sharply increases in the stationary phase of bacterial growth and also under a variety of stress conditions such as osmotic shock. We found out that the relative levels of AsdA remained the same under iron limitation and osmotic stress in *fur* and *rpoS* mutants. This raises the possibility of *asdA* expression being regulated by Fur and RpoS under conditions of iron limitation and osmotic stress respectively.

Pulse expression analysis was done to gain insight into the functional significances of AsdA. Results obtained indicate that overexpression of *asdA* led to a significant increase in *dnaA* mRNA level, implying that expression of the antisense strand of *dnaA* increases the stability of *dnaA* mRNAs which is likely to enhance their translation. This conclusion is further strengthened by the observation that *dnaA* mRNA levels shot up further when *asdA* was overexpressed in RNase III mutant background, which is the RNase likely to degrade DnaA/AsdA duplex. Although some antisense RNAs like AmgR [36] may cause a decrease in the levels of their target sense mRNA, many sense–antisense pairs exhibit positively co-regulated expression profiles that indicate a possible involvement of antisense RNAs in stabilizing *cis*-encoded mRNAs [30].

In conclusion, while we could not investigate the effect of the absence of AsdA on the target mRNA due to challenges involved in deleting a vital gene like *dnaA*, we have established by this study that antisense RNA of *dnaA* is indeed transcribed not merely as a by-product of the cell's transcription machinery but plays a vital role as far as stability of *dnaA* mRNA is concerned. This to the best of our knowledge becomes the first antisense RNA of *dnaA* reported to be directly associated with bacterial replication process.

## References

[1] Livny J, Waldor MK (2007) Identification of small RNAs in diverse bacterial species. Current Opinion in Microbiology 10: 96–101. 17383222 10.1016/j.mib.2007.03.005

[2] Waters LS, Storz G (2009) Regulatory RNAs in Bacteria. Cell 136: 615–628. 19239884 10.1016/j.cell.2009.01.043 PMC3132550

[3] Thomason KD, Storz G (2010) Bacterial antisense RNAs: How many are there and what are doing? Annu Rev Genet 44: 167–188. 20707673 10.1146/annurev-genet-102209-163523 PMC3030471

[4] Georg J, Wolfgang HR (2011) cis-Antisense RNA, Another Level of Gene Regulation in Bacteria. microbiology and molecular biology reviews 75: 286–300. 21646430 10.1128/MMBR.00032-10 PMC3122628

[5] Brantl S (2007) Regulatory mechanisms employed by cis-encoded antisense RNAs. Current Opinion in Microbiology 10: 102–109. 17387036 10.1016/j.mib.2007.03.012

[6] Aiso T, Murata M, Gamou S (2011) Transcription of an antisense RNA of a gadE mRNA is regulated by GadE, the central activator of the acid resistance system in *Escherichia coli* . Genes to Cells 16: 670–680. 21501346 10.1111/j.1365-2443.2011.01516.x

[7] Massé E, Salvail H, Desnoyers G, Arguin M (2007) Small RNAs controlling iron metabolism. Current Opinion in Microbiology 10: 140–145. 17383226 10.1016/j.mib.2007.03.013

[8] Hammer BK, Bassler BL (2007) Regulatory small RNAs circumvent the conventional quorum sensing pathway in pandemic *Vibrio cholerae* . Proceedings of the National Academy of Sciences 104: 11145–11149. 10.1073/pnas.0703860104 PMC1888797 17556542

[9] Lee E-J, Groisman EA (2010) An antisense RNA that governs the expression kinetics of a multifunctional virulence gene. Molecular Microbiology 76: 1020–1033. 20398218 10.1111/j.1365-2958.2010.07161.x PMC2909850

[10] Moon K, Gottesman S (2009) A PhoQ/P-regulated small RNA regulates sensitivity of *Escherichia coli* to antimicrobial peptides. Molecular Microbiology 74: 1314–1330. 19889087 10.1111/j.1365-2958.2009.06944.x PMC2841474

[11] Martínez LC, Yakhnin H, Camacho MI, Georgellis D, Babitzke P, et al (2011) Integration of a complex regulatory cascade involving the SirA/BarA and Csr global regulatory systems that controls expression of the *Salmonella* SPI-1 and SPI-2 virulence regulons through HilD. Molecular Microbiology 80: 1637–1656. 21518393 10.1111/j.1365-2958.2011.07674.x PMC3116662

[12] Sharma CM, Darfeuille F, Plantinga TH, Vogel J (2007) Small RNA regulates multiple ABC transporter mRNAs by targeting C/A-rich elements inside and upstream of ribosome-binding sites. Genes and development 21: 2804–2817. 17974919 10.1101/gad.447207 PMC2045133

[13] Papenfort K, Pfeiffer V, Mika F, Lucchini S, Hinton JCD, et al (2006) σE-dependent small RNAs of *Salmonella* respond to membrane stress by accelerating global omp mRNA decay. Molecular Microbiology 62: 1674–1688. 17427289 10.1111/j.1365-2958.2006.05524.x PMC1804206

[14] Gogol EB, Rhodius VA, Papenfort K, Vogel J, Gross CA (2011) Small RNAs endow a transcriptional activator with essential repressor functions for single-tier control of a global stress regulon. Proceedings of the National Academy of Sciences 108: 12875–12880. 10.1073/pnas.1109379108 PMC3150882 21768388

[15] Fröhlich KS, Papenfort K, Berger AA, Vogel J (2012) A conserved RpoS-dependent small RNA controls the synthesis of major porin OmpD. Nucleic Acids Research 40: 3623–3640. 22180532 10.1093/nar/gkr1156 PMC3333887

[16] Argaman L, Altuvia S (2000) fhlA repression by OxyS RNA: kissing complex formation at two sites results in a stable antisense-target RNA complex. Journal of Molecular Biology 300: 1101–1112. 10903857 10.1006/jmbi.2000.3942

[17] Schaper S, Messer W (1995) Interaction of the initiator protein DnaA of *Escherichia coli* with its DNA target. Journal of Biological Chemistry 270: 17622–17626. 7615570 10.1074/jbc.270.29.17622

[18] Mott ML, Berger JM (2007) DNA replication initiation: mechanisms and regulation in bacteria. Nature Reviews Microbiology 5: 343–354. 17435790 10.1038/nrmicro1640

[19] Messer W, Blaesing F, Jakimowicz D, Krause M, Majka J, et al (2001) Bacterial replication initiator DnaA. Rules for DnaA binding and rolesof DnaA in origin unwinding and helicase loading. Biochimie 83: 5–12. 11254968 10.1016/s0300-9084(00)01216-5

[20] Messer W, Weigel C (2003) DnaA initiator—also a transcription factor. Molecular microbiology 24: 1–6. 10.1046/j.1365-2958.1997.3171678.x 9140960

[21] Breier AM, Grossman AD (2009) Dynamic association of the replication initiator and transcription factor DnaA with the *Bacillus subtilis* chromosome during replication stress. Journal of bacteriology 191: 486–493. 19011033 10.1128/JB.01294-08 PMC2620820

[22] Katayama T, Ozaki S, Keyamura K, Fujimitsu K (2010) Regulation of the replication cycle: conserved and diverse regulatory systems for DnaA and *oriC* . Nature Reviews Microbiology 8: 163–170. 20157337 10.1038/nrmicro2314

[23] Datsenko KA, Wanner BL (2000) One-step inactivation of chromosomal genes in *Escherichia coli* K-12 using PCR products. Proc Natl Acad Sci U S A 97: 6640–6645. 10829079 10.1073/pnas.120163297 PMC18686

[24] Argaman L, Hershberg R, Vogel J, Bejerano G, Wagner EGH, et al (2001) Novel small RNA-encoding genes in the intergenic regions of *Escherichia coli* . Current Biology 11: 941–950. 11448770 10.1016/s0960-9822(01)00270-6

[25] Livak KJ, Schmittgen TD (2001) Analysis of relative gene expression data using real-time quantitative PCR and the 2(−Delta Delta C(T)) Method. Methods 25: 402–408. 11846609 10.1006/meth.2001.1262

[26] Viegas SC, Pfeiffer V, Sittka A, Silva IJ, Vogel J, et al (2007) Characterization of the role of ribonucleases in *Salmonella* small RNA decay. Nucleic acids research 35: 7651–7664. 17982174 10.1093/nar/gkm916 PMC2190706

[27] Raghavan R, Sloan DB, Ochman H (2012) Antisense Transcription Is Pervasive but Rarely Conserved in Enteric Bacteria. mBio 3. 10.1128/mBio.00156-12 PMC3419515 22872780

[28] Messer W (2002) The bacterial replication initiator DnaA. DnaA and *oriC*, the bacterial mode to initiate DNA replication. FEMS Microbiology Reviews 26: 355–374. 12413665 10.1111/j.1574-6976.2002.tb00620.x

[29] Padalon-Brauch G, Hershberg R, Elgrably-Weiss M, Baruch K, Rosenshine I, et al (2008) Small RNAs encoded within genetic islands of *Salmonella* Typhimurium show host-induced expression and role in virulence. Nucleic acids research 36: 1913–1927. 18267966 10.1093/nar/gkn050 PMC2330248

[30] Chinni SV, Raabe CA, Zakaria R, Randau G, Hoe CH, et al (2010) Experimental identification and characterization of 97 novel npcRNA candidates in *Salmonella enterica* serovar Typhi. Nucleic acids research 38: 5893–5908. 20460466 10.1093/nar/gkq281 PMC2943607

[31] Carpousis AJ, Luisi BF, McDowall KJ (2009) Endonucleolytic Initiation of mRNA Decay in *Escherichia coli* . Progress in Molecular Biology and Translational Science 85: 91–135. 19215771 10.1016/S0079-6603(08)00803-9

[32] Regnier P, Arraiano CM (2000) Degradation of mRNA in bacteria: emergence of ubiquitous features. Bioessays 22: 235–244. 10684583 10.1002/(SICI)1521-1878(200003)22:3<235::AID-BIES5>3.0.CO;2-2

[33] Selinger DW, Cheung KJ, Mei R, Johansson EM, Richmond CS, et al (2000) RNA expression analysis using a 30 base pair resolution *Escherichia coli* genome array. Nature biotechnology 18: 1262–1268. 10.1038/82367 11101804

[34] Sharma CM, Hoffmann S, Darfeuille F, Reignier J, Findeiß S, et al (2010) The primary transcriptome of the major human pathogen *Helicobacter pylori* . Nature 464: 250–255. 20164839 10.1038/nature08756

[35] Moon K, Gottesman S (2009) A PhoQ/P-regulated small RNA regulates sensitivity of *Escherichia coli* to antimicrobial peptides. Mol Microbiol 74: 1314–1330. 19889087 10.1111/j.1365-2958.2009.06944.x PMC2841474

[36] Lee EJ, Groisman EA (2010) An antisense RNA that governs the expression kinetics of a multifunctional virulence gene. Molecular microbiology 76: 1020–1033. 20398218 10.1111/j.1365-2958.2010.07161.x PMC2909850

[37] Johansen J, Eriksen M, Kallipolitis B, Valentin-Hansen P (2008) Down-regulation of outer membrane proteins by noncoding RNAs: unraveling the cAMP-CRP- and sigmaE-dependent CyaR-ompX regulatory case. J Mol Biol 383: 1–9. 18619465 10.1016/j.jmb.2008.06.058

[38] Mutalik VK, Nonaka G, Ades SE, Rhodius VA, Gross CA (2009) Promoter strength properties of the complete sigma E regulon of *Escherichia coli* and *Salmonella enterica* . J Bacteriol 191: 7279–7287. 19783623 10.1128/JB.01047-09 PMC2786571

[39] Papenfort K, Pfeiffer V, Mika F, Lucchini S, Hinton JC, et al (2006) SigmaE-dependent small RNAs of *Salmonella* respond to membrane stress by accelerating global omp mRNA decay. Mol Microbiol 62: 1674–1688. 17427289 10.1111/j.1365-2958.2006.05524.x PMC1804206

[40] Huang X, Phung L, Dejsirilert S, Tishyadhigama P, Li YH, et al (2004) Cloning and Characterization of the gene encoding the z66 antigen of *Salmonella* enterica serovar Typhi. FEMS Microbiol Lett 234: 239–246. 15135528 10.1016/j.femsle.2004.03.030

[41] Du H, Wang M, Luo Z, Ni B, Wang F, et al (2011) Coregulation of gene expression by sigma factors RpoE and RpoS in *Salmonella enterica* serovar Typhi during hyperosmotic stress. Curr Microbiol 62: 1483–1489. 21311887 10.1007/s00284-011-9890-8

